# Volume XXIX.—Finis

**Published:** 1890-07

**Authors:** 


BUFFALO MEDICAL AND SURGICAL JOURNAL
A MONTHLY REVIEW OF MEDICINE AND SURGERY.

*EDITORS:*
THOS. LOTHROP, M. D. - - - W. W. POTTER, M. D

All communications, whether of a literary or business character, should be addressed to the editors: 284 FRANKLIN STREET, BUFFALO, N. Y.

### Editorial.

### VOLUME XXIX.—FINIS.

WITH this number the twenty-ninth volume of the new series of this magazine comes to an end, and this would appear a proper time in which to indulge in retrospection.

With the beginning of the volume we made some changes in the make-up of the JOURNAL that we think have resulted in substantial gain to our patrons. These were mainly in increasing to sixty-four pages monthly, in the adoption of long primer type as a standard, in printing reports of cases and long extracts in brevier, and in the numerous illustrations of superior artistic merit that have appeared from time to time. The half-tones from the celebrated house of Matthews, Northrup & Co., of this city, are especially worthy of mention.

The list of contributors to this volume is pointed to with pride, as indicating the high order of literary and professional excellence of our original department, and the society reports are well worthy of mention in connection with the original work that has first seen the light of publication through these pages.

In order to maintain this standard, which we purpose doing in the next volume, it is necessary that a considerable expenditure of money must be made, and we ask our subscribers to remit promptly the sums due on their accounts. The price of the JOURNAL will remain the same—two dollars a year—to advance paying subscribers; all others will be charged $2.50 a year.

To all new subscribers who will send us $3.00 we will send the JOURNAL and the *Cosmopolitan Magazine* for one year. This is an offer that should prove tempting to any one familiar with good literature. The *Cosmopolitan* takes rank with the best of American monthlies, and is second to none in the world. It has only to be seen to be appreciated, and it cannot be obtained in this combination much longer.

